# Two Cases of Thoracentesis

**Published:** 1866-09

**Authors:** J. W. Freer

**Affiliations:** Prof. of Physiology and Surg. Anatomy in Rush Med. College


### CHICAGO MEDICAL JOURNAL.

Vol. XXIII. SEPTEMBER, 1866. No. 9.

### ORIGINAL CONTRIBUTIONS.

*Two Cases of Thoracentesis — Recovery.* By J. W. Freer, M. D., Prof. of Physiology and Surg. Anatomy in Rush Med. College.

Case I. B. Smith, æt. 12 years, came under my observation through the kindness of Dr. Dodge, the attending physician, on the 12th of February, 1860. The early history of the case is quite obscure, the boy having been sick with what he termed a pain in his side some five weeks before applying for medical aid, at which time the Doctor informed me the left pleural cavity was completely distended with fluid.

*Symptoms at the time of operation, Feb. 12th.*—Left pleural cavity greatly distended; intercostal spaces pressed out even with the ribs; measurement 1¾ inches greater than the right side; heart occupied a median position; dullness on percussion throughout the diseased side; no respiratory murmur; strong puerile respiration over the right lung. The dyspnœa was very urgent, attended with livid countenance, blueness of lips and orthopnœa.

The operation of thoracentesis was at once decided upon and performed between the sixth and seventh ribs, at the junction of the digitations of the serratus magnus with the external oblique muscles. More than 70 ounces of pus were withdrawn.

As I did not see the case again, I will refer to Dr. Dodge for the subsequent history.

He says: "I had an opportunity of watching the case more than six months after the operation. In four weeks he was running about the street. From time to time, on examining his chest, I found the respiratory murmur in the compressed lung continued to increase and improve, until finally the function of the lung seemed quite restored and the boy was enjoying robust health."

CASE II. John Kennedy, aged 23, gas maker by occupation, of robust constitution and sanguine temperament. Saw him the first time on February 9th, 1866, in consultation with Dr. Dodge. The history of the case was that of pleuritis of left side with subsequent effusion.

*Symptoms.*—No respiratory movements of left thorax; no resonance on percussion excepting in the sub-clavicular region; in the sitting posture, respiratory murmur absent; strong puerile respiration on the right side. The dyspnœa was of the most urgent character; the lips blue and countenance livid; number of respirations per minute, 40.

The patient was obliged to maintain the sitting posture in order to alleviate these distressing symptoms.

Thoracentesis was at once decided upon, and the operation performed in the same situation as in the former case. About 90 ounces of clear serum were withdrawn, with immediate relief of the most urgent symptoms. The lung did not fully expand at once, but bronchial respiration could be heard over the anterior and upper two-thirds of the diseased side. I did not see the patient again, but Dr. Dodge informs me that, at the end of four weeks, he seemed to have fully recovered, and at the present time is engaged in his usual pursuits.

*Remarks.*—Respecting the propriety of tapping the chest in cases of empyema, we believe at the present time there is no difference of opinion among enlightened medical men—all concede it to be an operation about which there need be no hesitation; and had not the recovery in this case been so unusual as regards time and final completeness, I would not have troubled the readers of the Journal with its rehearsal. In fact, recovery from empyema, with or without operation, is the exception rather than the rule.

Bowditch says: "In 26 out of 75 cases, serum was found, and 21 of these patients got wholly well. If after the first operation the fluid becomes purulent, an almost fatal prognosis should be made."

Again, he says: "Pus was found at the first operation in 24 cases. Seven of these cases recovered wholly; 7 died; 9 were relieved one or many times; but they had either a long and tedious illness, terminating usually in phthisis, or a fistulous opening, or a still doubtful result."

As regards the second case, that of serous effusion from pleuritic inflammation, the propriety of performing the operation of paracentesis is not as fully established, unless as a dernier resort. We are directed in such cases to exhaust every other means for the purpose of promoting absorption—such as blisters, iodine, mercury, etc.; these failing, and the danger having become imminent, we are permitted to tap. But thanks to the labors of Bowditch, Wyman and others, many of the fancied dangers attending this operation have been resolved into groundless apprehension. The principal danger, that of admitting air, is entirely obviated by the use of Wyman's suction pump, or by using a trocar and canula with a stopcock, and attaching to the instrument a rubber tube or a gum catheter, the distal end of which is immersed in water.

With the above precautions, we believe it should be resorted to before urgent symptoms appear, while the lung is still free to expand, and not bound down by adhesions or otherwise injured in its textures.

